# High prevalence of *Clonorchis sinensis* infections and coinfection with hepatitis virus in riverside villages in northeast China

**DOI:** 10.1038/s41598-020-68684-x

**Published:** 2020-07-16

**Authors:** Yanhang Gao, Yanqing Li, Xiaowen Liu, Tong Zhang, Ge Yu, Yang Wang, Ying Shi, Xiumei Chi, Xiaomei Wang, Xiuzhu Gao, Ruihong Wu, Yingyu Zhang, Lei Hang, Shijuan Sun, Yazhe Guan, Ying Xu, Jing Meng, Xu Liu, Chang Jiang, Heming Ma, Liting Luo, Qi Yan, Xin Yin, Fei Peng, Yixiao Zhi, Weige Qu, Xia Zhang, Tianqi Ren, Lili Liu, Jinming Zhao, Feiyu Zhang, Adila Yakepu, Yu Pan, Hongqin Xu, Junqi Niu

**Affiliations:** 1 Department of Hepatology, The First Hospital of Jilin University, Jilin University, No. 71 Xinmin Street, Changchun, 130021 China; 2 Jilin Province Key Laboratory of Infectious Diseases, Laboratory of Molecular Virology, No. 519 Dongminzhu Street, Changchun, 130061 China

**Keywords:** Gastroenterology, Health care

## Abstract

In China, the prevalence of *Clonorchis sinensis *(*C. sinensis*) infections is only evaluated at the provincial level by national sampling surveys, and data from villages and counties are still lacking. In this study, we conducted a cross-sectional survey in 10 villages located along the Lalin River in northeast China. Clonorchiasis was diagnosed using a modified Kato–Katz method that detects the *C. sinensis* egg in stools. A total of 3,068 persons were screened and 2,911 were recruited for the study. Overall, the prevalence of *C. sinensis* infection was 29.3%. Among 175 participants who were cured after antiparasitic treatment, 54 (30.86%) were re-infected in this survey. After calibration of potential confounders, male gender, occupation as a farmer, smoking, and occasionally or frequently eating raw fish were independent risk factors for *C. sinensis* infection. The results of laboratory examinations in the *C. sinensis*/hepatitis B or C virus co-infection group were similar to those in the hepatitis B or C virus mono-infection groups. In conclusion, *C. sinensis* is highly endemic in villages along the Lalin River, and the primary route of infection is the consumption of raw freshwater fish. Co-infection with *C. sinensis* did't aggravate the clinical manifestations of viral hepatitis in this cross-sectional study.

## Introduction

*Clonorchis sinensis* (*C*. *sinensis*) is an important food-borne zoonotic parasite that has infected approximately 15 million people worldwide; countries in eastern and Southeast Asia, including China, Japan, Korea, and Vietnam, account for a large proportion of infections^1-4^. There are 13 million people infected with *Clonorchis sinensis* in China, which is the country with the largest number of infections in the world^5,6^. Three large-scale clonorchiasis investigations have been conducted in mainland China. The first national parasite investigation, which covered 30 provinces/municipalities/autonomous regions (P/M/As) from 1988 to 1992 (hereinafter referred to as 1992), showed that the prevalence of clonorchiasis was 0.37%^7^. The prevalence increased to 0.58% in the second national parasite investigation, which included 31 P/M/As from 2001 to 2004^8^. Another special clonorchiasis investigation in 27 endemic P/M/As was conducted during the years 2001–2004 and showed that the prevalence was 2.40%, with 12.49 million people infected^8,9^. Jilin Province is one of the major endemic regions in China^8^. The prevalence of *Clonorchis sinensis* infections is only evaluated at the provincial level by national sampling surveys in China, and data from villages and counties are still lacking. The current study results supplement *C*. *sinensis* infection data in rural areas along rivers in Jilin Province.

Persistent and chronic infections of *C*. *sinensis* often cause the development and progression of hepatobiliary diseases, such as cholangitis, cholelithiasis, cholecystitis, pancreatitis, hepatic fibrosis, liver cancer, and cholangiocarcinoma (CCA)^10^. Cholangiocarcinoma is the most severe complication of *C. sinensis* infection^11,12^. Although great progress has been made in the treatment of cholangiocarcinoma in recent years, 5-year survival rates remains very low^13^. A Korean study reported that *C. sinensis* infection causes 25% of CCA cases in endemic areas; approximately one-tenth of CCA cases are estimated to be caused by *C. sinensis* infection, and the risk of CCA in areas hyper-endemic for *C. sinensis* is higher than in other areas^14^. However, the cause of most cholangiocarcinomas is still not identified. Recently, researchers have reported that CCA may be caused by viral hepatitis B and C or hepatolithiasis cirrhosis^15,16^. Similar to *C. sinensis* infection, the prevalence rates of hepatitis B virus (HBV) and hepatitis C virus (HCV) infections are also considerably high in China. HBV, HCV and *C. sinensis* target the same organ, but little is known about whether co-infection can aggravate symptoms, and current data regarding whether there is any association between co-infection and hepatocellular carcinoma or cholangiocarcinomas are limited.

This cross-sectional study obtained baseline data for our long-term follow-up observation in areas highly endemic for *C. sinensis*. It is expected that the follow-up data of *C. sinensis* infection and cholangiocarcinoma cases will aid in the evaluation of the disease burden of *C. sinensis* infection and ultimately promote *C. sinensis* prevention. We also focused on whether *C. sinensis* co-infection affected the disease state of HBV and HCV infections. We look forward to clarifying the impact of hepatitis virus on the occurrence and development of cholangiocarcinoma in a long-term follow-up study.

## Results

### Demographic characteristics and prevalence of C. sinensis infection

Of the 3,068 participants who participated in the survey, 2,911 (male, 1,496 [51.4%]; female, 1,415 [48.6%]) participants were enrolled in the study. 157 participants were excluded due to incomplete or unreliable information. Out of 2,911 study participants, 2,056 (70.6%) were aged between 31 and 60 years. There were 1,496 (51.4%) males and 1,415 (48.6%) females. In all, the prevalence of subjects infected with C. sinensis was 29.3% (95% CI: 27.6%-31.0%). In Table 1, the positive rates of C. sinensis infection varied markedly in the different age groups. The highest C. sinensis positivity rate(30.7%) was in group aged 31–60 years, whereas subjects under 30 years had the lowest prevalence rate (16.8%) of C. sinensis-positive results. Furthermore, gender differences in the *C*. *sinensis* positivity rates were observed, with higher rates found in males (39.4%) than in females (18.7%). Among 2,911 individuals, 2,759 tests included assays for detecting serum HCV antibodies and HBV serological markers. After 152 cases without HBV and HCV tests were excluded, 165 (5.99%) of the remaining 2,759 participants were hepatitis B surface antigen (HBsAg) positive, and 850 (30.8%) of 2,759 participants were HCV antibody positive.

**Table 1:** Univariate and multivariate analysis of socio-demographic characteristics by *Clonorchis sinensis* infection among residents in riverside villages.

Variable	Total N	Clonorchiasis + N (%)	Univariate analysis		Multivariate analysis		
			Odds ratio (95% CI)	*P* value	OR	95% CI for OR	*P* value
Total	2,911	854 (29.3)	–	–	–	–	–
**Sex**							
Female	1,415	265 (18.7)	–	–	Reference	Reference	Reference
Male	1,496	589 (39.4)	2.818 (2.380–3.337)	< 0.001	1.95	1.58–2.40	< 0.001
**Age**							
≤ 30 years old	95	16 (16.8)	–	0.005	–	–	–
31–60	2056	631 (30.7)	2.186 (1.267–3.772)	0.004	–	–	–
˃ 60 years old	760	207 (27.2)	1.848 (1.055–3.237)	0.03	–	–	–
**Education**							
College	167	29 (17.4)	–	< 0.001	–	–	–
Middle school	1,015	339 (33.4)	2.386 (1.566–3.636)	< 0.001	–	–	–
Primary school	1729	486 (28.1)	1.861 (1.230–2.814)	0.003	–	–	–
**Occupation**							
Non-farmer	278	49 (17.6)	–	–	Reference	Reference	Reference
Farmer	2,633	805 (30.6)	2.058 (1.495–2.833)	0.000	2.31	1.66–3.22	< 0.001
**Cigarette smoking**							
No	1675	432 (25.8)	–	0.000	Reference	Reference	Reference
Yes	1,092	384 (35.2)	1.561 (1.322–1.842)	< 0.001	1.1	0.92–1.32	0.302
Quit smoking	144	38 (26.4)	1.031 (0.701–1.518)	0.875	0.68	0.45–1.03	0.069
**Alcohol consumption**							
No	1985	463 (23.3)	–	0.000	Reference	Reference	Reference
Yes	816	352 (43.1)	2.494 (2.097–2.965)	0.000	1.31	1.06–1.63	0.013
quit drinking	110	39 (35.5)	1.806 (1.205–2.705)	0.004	1.11	0.71–1.73	0.649
**Eating raw fish**							
Never	1,049	154 (14.7)	–	0.000	Reference	Reference	Reference
Occasionally	1787	660 (36.9)	3.403 (2.797–4.141)	0.000	2.58	2.10–3.18	< 0.001
Frequently	75	40 (53.3)	6.642 (4.090–10.785)	0.000	3.93	2.38–6.51	< 0.001
**Reared cats and dogs as pets or guardians**							
No	1928	544 (28.2)	–	–	–	–	–
Yes	983	310 (31.5)	1.172 (0.992–1.385)	0.063	–	–	–
**HBV infection**							
No	2,594	753 (29.0)	–	0.039	–	–	–
Yes	165	62 (37.6)	1.471 (1.062–2.039)	0.02	–	–	–
Unclear	152	39 (25.7)	0.844 (0.581–1.226)	0.373	–	–	–
**HCV infection**							
No	1909	578 (30.3)	–	0.263			
Yes	850	237 (27.9)	0.890 (0.745,1.06)	0.203			
Unclear	152	39 (25.7)	0.795 (0.545,1.159)	0.231			

### Univariate analysis of variables associated with C. sinensis infection

The results of the univariate comparisons are presented in Table 1. The results revealed that 1,862 (64.0%) participants reported eating raw fish. Most of them ate raw fish less than 2 times per week, and only 75 (4.0%) of them ate raw fish frequently (more than 2 times per week). Participants with a habit of eating raw fish were more likely to have *C. sinensis* infection than those who did not eat raw fish; the higher the frequency was, the greater the risk. Male gender (OR = 2.818, 95% CI: 2.380–3.337), older age (OR = 2.186, 95% CI: 1.267–3.772; OR = 1.848, 95% CI: 1.055–3.237), a low literacy level (OR = 2.386, 95% CI: 1.566–3.636; OR = 1.861, 95% CI: 1.230–2.814), cigarette smoking (OR = 1.561, 95% CI: 1.322–1.842), alcohol consumption (OR = 2.494, 95% CI: 2.097–2.965; OR = 1.806, 95% CI: 1.205–2.705) and occupation as a farmer (OR = 2.058, 95% CI: 1.495–2.833) were significantly associated with an increased risk of *C. sinensis* infection.

### Multivariate analysis of variables associated with C. sinensis infection

Age, gender, occupation, education, cigarette smoking, alcohol consumption, HBV infection and eating raw fish, which were associated with *C. sinensis* infection in univariate analysis, were included in the multiple logistic regression model. After multivariate correction, male gender (OR = 1.95, *P* < 0.001), occupation as a farmer (OR = 2.31, *P* < 0.001), alcohol consumption (OR = 1.31, *P* = 0.013), and eating raw fish occasionally (OR = 2.58, *P* < 0.001) and frequently (OR = 3.93, *P* < 0.001) were independently associated with *C. sinensis* infection in the multivariate analysis (Table 1). Eating raw fish had the most significant associations with *C. sinensis* infection, and the odds ratios indicated that eating raw fish frequently (OR = 6.642, 95% CI:4.090–10.785) were at highest risk for *C. sinensis* infection. Age, cigarette smoking and HBV infection were not found to be significantly associated with *C. sinensis* infection in this study.

### *C. sinensis*/HBV or *C. sinensis*/HCV co-infection

Among 854 participants with *C. sinensis* infection (*C. sinensis* eggs in stools), 46 were co-infected with HBV (HBsAg positive), 60 were co-infected with HCV (HCV-RNA positive) and 1 was co-infected with HBV and HCV (HBsAg positive and HCV-RNA positive). Age and gender were used to match co-infected and mono-infected individuals. Compared with the *C. sinensis* mono-infection group, the AST to platelet ratio index(APRI) levels in the HBV co-infection and **HBV** mono-infection group were significantly higher (*P* = 0.049). The HBV/*C. sinensis* co-infection group had a higher eosinophilic granulocyte level than the HBV mono-infection group. There is no significant difference of other indicators in the three groups (Table 2). The baseline characteristics of the HCV/*C. sinensis* co-infection group are presented in Table 3. The HCV/*C. sinensis* co-infection group had similar alanine aminotransferase (ALT), aspartate aminotransferase (AST), alkaline phosphatase (ALP), gamma-glutamyl transpeptidase (GGT), albumin (ALB), glucose (GLU) and total platelet (PLT) levels and AST to PLT ratio index (APRI) and fibrosis-4 scores (FIB-4) compared to those of the HCV mono-infected group. ALT, AST, total protein (TP), total bilirubin (TBIL), indirect bilirubin (IBIL), GGT, ALP, APRI and FIB-4 scores were significantly higher in the co-infection group than in the *C. sinensis* mono-infected group. The differences between the two groups were statistically significant. The albumin to globulin (A/G), triglyceride (TG), cholesterol, low-density lipoprotein (LDL), white blood cell (WBC), and PLT levels were higher in the mono-infection group than in the co-infection group. The differences were statistically significant.

**Table 2:** Baseline characteristics of HCV/*C. sinensis* co-infection group and HCV or *C. sinensis* mono-infection groups.

	Hepatitis B (n = 46)	HBV/*C. sinensis* coinfection (n = 46)	*C. sinensis* (n = 46)	*P*1	*P*2
Age (years, mean ± SD)	50.59 ± 10.01	50.65 ± 9.81	50.63 ± 9.76	0.999	0.999
Sex (Male, %)	31 (67.4)	31 (67.4)	31 (67.4)	1.000	1.000
ALT (IU/mL)	41.0 (34.3, 49.5)	38.5 (32.8, 51.0)	39.5 (30.5, 49.3)	0.56	0.912
AST (IU/mL)	33.0 (26.0, 42.5)	32.5 (27.8, 39.0)	29.5 (25.0, 40.3)	0.922	0.137
Total protein (g/L)	78.0 (72.8, 84.3)	77.0 (72.0, 83.3)	78.5 (72.0, 81.3)	0.46	0.587
ALB (g/L)	44.5 (41.0, 48.3)	44.0 (41.0, 47.0)	44.5 (42.0, 46.0)	0.45	0.543
ALB/GLB	1.40 (1.08, 1.52)	1.36 (1.19, 1.54)	1.34 (1.17, 1.59)	0.934	0.936
TBIL (µmol/L)	18.0 (13.0, 23.0)	18.0 (14.0, 22.0)	17.0 (13.8, 21.0)	0.745	0.766
IBIL (µmol/L)	11.0 (6.75, 16.0)	9.0 (7.0, 14.0)	10.0 (6.0, 13.0)	0.484	0.829
GGT (U/L)	28.5 (18.8, 59.5)	31.5 (21.8, 74.3)	33.5 (24.8, 65.8)	0.292	0.614
ALP (U/L)	69.0 (50.0, 83.0)	61.5 (45.5, 72.3)	67.0 (53.8, 79.5)	0.184	0.183
Creatinine (μmol/L)	62.0 (54.5, 73.0)	64.5 (58.0, 70.5)	63.5 (55.5, 75.3)	0.763	0.699
BUN (mmol/L)	5.1 (4.4, 5.9)	5.25 (4.50, 6.03)	5.5 (4.6, 6.2)	0.716	0.39
Uric acid (µmol/L)	354 (304, 435)	362.0 (308.3, 422.8)	360.0 (311.5, 435.3)	0.679	0.885
TG (mmol/L)	1.34 (0.95, 1.83)	1.52 (1.11, 2.20)	1.79 (1.19, 2.91)	0.226	0.328
Cholesterol (mmol/L)	3.45 (2.80, 3.88)	3.60 (3.10, 4.30)	3.90 (3.30, 4.50)	0.088	0.148
LDL (mmol/L)	1.23 (0.91, 1.56)	1.40 (0.88, 2.02)	1.45 (1.12, 2.08)	0.517	0.293
GLU (mmol/L)	3.40 (3.10, 3.55)	3.80 (3.30, 4.90)	3.50 (3.25, 4.15)	0.388	0.873
WBC (*109/L)	6.96 (6.22, 8.27)	6.78 (5.75, 7.90)	6.73 (5.81, 7.63)	0.571	0.932
RBC (*1,012/L)	4.64 (4.23, 5.15)	4.64 (4.16, 5.00)	4.67 (4.40, 4.97)	0.363	0.679
PLT (*109/L)	159.5 (133.8, 190.0)	161.0 (129.8, 198.0)	178.5 (145.8, 216.5)	0.767	0.135
Lymphocyte (*109/L)	2.27 (1.89, 3.04)	2.41 (1.95, 3.04)	2.38 (1.96, 2.71)	0.87	0.582
Neutrophil (*109/L)	3.75 (3.27, 4.22)	3.51 (2.80, 4.30)	3.29 (2.90, 4.26)	0.461	0.746
Eosinophil count (*109/L)	0.15 (0.11, 0.21)	0.18 (0.12, 0.34)	0.19 (0.12, 0.42)	0.509	0.673
Haemoglobin (g/L)	153.5 (139.0, 169.3)	155.5 (134.5, 167.0)	152.5 (144, 160)	0.54	0.749
lg10 (HBV-DNA)	3.39 (2.30, 4.63)	2.91 (2.36, 5.60)	–	1	–
APRI	0.45 (0.31, 0.62)	0.47 (0.33, 0.65)	0.38 (0.28, 0.47)	0.904	0.049
FIB-4	1.70 (1.12, 2.44)	1.65 (1.08, 2.69)	1.28 (1.02, 1.80)	0.907	0.082

*ALT* alanine aminotransferase, *AST* aspartate aminotransferase, *ALB* albumin, *GLB* globulin, *ALB/GLB* albumin to globulin, *TBIL* total bilirubin, *IBIL* indirect bilirubin, *GGT* gamma-glutamyl transpeptidase, *ALP* alkaline phosphatase, *BUN* blood urea nitrogen, *TG* triglyceride, *LDL* low-density lipoprotein, *GLU* glucose, *WBC* white blood cell, *RBC* red blood cell, *PLT* platelet, APRI, levels and AST to PLT ratio index, *FIB-4* fibrosis-4 scores.

**Table 3:** Baseline characteristics of HBV/*C. sinensis* co-infection group and HBV or *C. sinensis* mono-infection groups.

	Hepatitis C (n = 59)	HCV/*C. sinensis* coinfection (n = 59)	*C. sinensis* (n = 59)	*P*1	*P*2
Age (years, mean ± SD)	57.78 ± 7.91	57.54 ± 8.47	57.46 ± 8.33	0.876	0.956
Sex (Male, %)	46 (78.0)	46 (78.0)	46 (78.0)	1.000	1.000
ALT (IU/mL)	54.0 (41.0, 81.0)	62.0 (42.0, 103.0)	38.0 (31.0, 48.0)	0.236	< 0.001
AST (IU/mL)	45.0 (32.0, 67.0)	54.0 (38.0, 87.0)	28.0 (24.0, 35.0)	0.052	< 0.001
Total protein (g/L)	78.0 (72.0, 85.0)	79.0 (75.0, 85.0)	77.0 (72.0, 82.0)	0.292	0.031
ALB (g/L)	43.0 (41.0, 45.0)	43.0 (42.0, 45.0)	44.0 (42.0, 46.0)	0.184	0.539
ALB/GLB	1.22 (1.07, 1.43)	1.18 (1.09, 1.35)	1.36 (1.23, 1.53)	0.51	0.001
TBIL (µmol/L)	17.0 (12.0, 22.0)	19.0 (14.0, 24.0)	16.0 (13.0, 20.0)	0.164	0.036
IBIL (µmol/L)	10.0 (6.0, 13.0)	11.0 (7.0, 15.0)	9.0 (6.0, 12.0)	0.111	0.025
GGT (U/L)	53.0 (29.0, 104.0)	69.0 (37.0, 143.0)	36.0 (25.0, 69.0)	0.114	0.001
ALP (U/L)	73.0 (61.0, 93.0)	78.0 (61.0, 93.0)	70.0 (56.0, 81.0)	0.577	0.035
Creatinine (μmol/L)	65.0 (58.0, 73.0)	65.0 (56.0, 75.0)	66.0 (58.0, 74.0)	0.732	0.724
BUN (mmol/L)	5.5 (4.5, 6.6)	5.80 (4.60, 6.60)	5.5 (4.8, 6.2)	0.628	0.554
Uric acid (µmol/L)	363.0 (330.0, 404.0)	377.0 (315.0, 424.0)	365.0 (305.0, 438.0)	0.317	0.844
TG (mmol/L)	1.38 (0.85, 1.73)	1.28 (0.89, 2.04)	1.54 (1.19, 2.54)	0.861	0.020
Cholesterol (mmol/L)	3.30 (2.80, 3.70)	3.30 (2.90, 4.00)	3.80 (3.20, 4.40)	0.425	0.003
LDL (mmol/L)	1.22 (0.91, 1.68)	1.27 (0.87, 1.77)	1.66 (1.16, 2.23)	0.998	0.011
GLU (mmol/L)	3.85 (3.53, 4.05)	4.10 (3.60, 4.40)	3.70 (3.35, 6.48)	0.377	0.535
WBC (*109/L)	6.28 (4.82, 7.73)	6.02 (5.12, 7.30)	6.81 (5.83, 8.09)	0.72	0.012
RBC (*1,012/L)	4.49 (4.28, 4.97)	4.57 (4.18, 4.93)	4.64 (4.34, 4.85)	0.901	0.825
PLT (*109/L)	144.0 (102.0, 179.0)	136.0 (106.0, 176.0)	180.0 (147.0, 215.0)	0.677	< 0.001
Lymphocyte (*109/L)	2.12 (1.73, 2.68)	2.00 (1.73, 2.49)	2.30 (1.97, 2.53)	0.62	0.029
Neutrophil (*109/L)	3.28 (2.29, 4.66)	3.30 (2.63, 4.03)	3.62 (2.88, 4.40)	0.733	0.039
Eosinophil count (*109/L)	0.15 (0.09, 0.20)	0.17 (0.11, 0.28)	0.25 (0.16, 0.47)	0.177	0.004
Haemoglobin (g/L)	152.0 (144.0, 161.0)	153.0 (142.0, 164.0)	153.0 (145.0, 161.0)	0.784	0.981
lg (HCV-RNA)	6.43 (5.71, 6.92)	6.27 (5.49, 6.82)	–	0.587	–
HCV-Genotype (1b, %)	32 (54.2)	27 (45.8)	–	0.361	–
APRI	0.74 (0.49, 1.47)	0.88 (0.55, 1.88)	0.35 (0.28, 0.52)	0.156	< 0.001
FIB-4	2.54 (1.65, 4.22)	3.01 (1.93, 5.39)	1.52 (1.18, 2.08)	0.188	< 0.001

*ALT* alanine aminotransferase, *AST* aspartate aminotransferase, *ALB* albumin, *GLB* globulin, *ALB/GLB* albumin to globulin, *TBIL* total bilirubin, *IBIL* indirect bilirubin, *GGT* gamma-glutamyl transpeptidase, *ALP* alkaline phosphatase, *BUN* blood urea nitrogen, *TG* triglyceride, *LDL* low-density lipoprotein, *GLU* glucose, *WBC* white blood cell, *RBC* red blood cell, *PLT* platelet, APRI, levels and AST to PLT ratio index, *FIB-4* fibrosis-4 scores.

### Repeated infection

**Figure 1:**
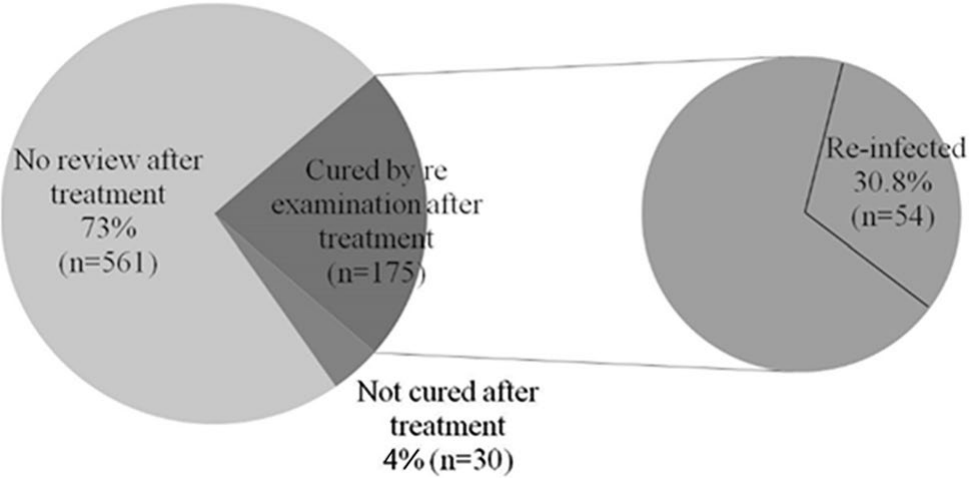
*Clonorchis sinensis* re-infection rate in this survey. Among 766 people who received quinolone treatment, 175 were cured after treatment, 561 were not reviewed, and 30 were not cured at one-month follow-up. Among the 175 people who were cured after the first treatment, 54 people were re-infected, accounting for 30.86%.

Praziquantel is the recommended drug for the treatment of clonorchiasis^17^. Since local disease control often distributes local therapeutic drugs, among our participants, 766 people received quinolone treatment (25 mg/kg praziquantel thrice daily for 1 or 2 consecutive days); 175 were cured after treatment, 561 were not reviewed, and 30 were not cured. Among the 175 people who were cured after the first treatment, this study found that 54 people were re-infected, accounting for 30.86% (Fig. 1).

## Discussion

In this study, the prevalence of *C. sinensis* infection in riverside villages in Fuyu City was 29.3%, which is tenfold higher than the prevalence of *C. sinensis* infection in the known endemic areas of China (2.4%)^8^. Our results showed that the prevalence of *C. sinensis* infection was higher in males than in females. This is because males are more likely to eat raw fish and shrimp than are females. A similar difference in the prevalence of *C*. *sinensis* infection between men and women has been shown in previous *C. sinensis* infection investigations in China from 2002 to 2004^8^. Infection was identified in all age groups, with the highest prevalence in the age group of 31–60 years (30.7%). These results are similar to others, which indicated that adults had high infection rates in certain provinces in which the main infection method was raw fish consumption. Regarding occupation, there was a higher prevalence among farmers (30.6%, 805/2,633) than among non-farmers (17.6%, 49/278). This may be related to a relatively low literacy level and lack of personal hygiene knowledge in farmers. For example, many farmers use water directly from the river as domestic or drinking water, which may increase the chance of *C. sinensis* infection. Further, the farmers in our study were likely to have unhealthy dietary habits, which has been reported in other studies^18^. Our previous study reported that the prevalence of HCV infection was high among residents in Fuyu^19^. There are many HCV and *C. sinensis* co-infections in Fuyu, and the potential interaction between the two diseases has not received wide attention. Trepo C et al. reported that hepatitis B infection is highly prevalent in many clonorchiasis-endemic areas^20^. In the present study, we also found that *C. sinensis* infection was highly prevalent in HBsAg-positive participants (37.6% vs. 29.0%), which confirms the above results.

In the multivariate model, independent risk factors associated with *C. sinensis* infection were male gender, occupation as a farmer, drinking alcohol and eating raw fish. The risk factor most strongly associated with *C. sinensis* infection was eating raw fish, which supports the hypothesis that eating raw fish was an important predisposing factor for the establishment of a large reservoir of *C. sinensis* infection in Fuyu City. People who habitually eat raw or undercooked fish and eat outside frequently have a higher infection risk ratio than those who do not. In our findings, a greater proportion of men have this unhealthy eating habit than women, as do farmers compared to people in other occupations, which is similar to other studies^21,22^. In the present study, alcohol consumption was significantly associated with an increased risk of *C. sinensis* infection in the multivariate analysis, and this result was consistent with another report^23^ since the local residents drank alcohol while eating raw fish and shrimp. Additionally, more males (50.3%) drank while eating raw fish in comparison with females (5%, *P* = 0.000); thus, this outcome could have resulted from the influence of gender. This finding reflects Chinese culture, in which it is more common for men than for women to drink alcohol. In addition, many people mistakenly think that alcoholic drink can kill bacteria and parasites in the food, and so they are not afraid of being infected by pathogens from potentially contaminated food. This also explains why drinking alcohol is one of the risk factors for liver fluke infection.

Our previous study found that Fuyu City is highly endemic for hepatitis C^19^. This study also confirms that result, and among 854 *C. sinensis*-infected participants, 237 (27.8%) were anti-HCV positive and 59 (6.9%) were HCV-RNA positive. We compared the results of laboratory examinations between the co-infection group and mono-infection group and found that the results of laboratory examinations in the *C. sinensis*/HBV group were similar to those in the *C. sinensis* and HBV mono-infection groups. Xu's study indicated that the *C*. *sinensis*/HBV co-infection group presented decreased liver function and increased HBV DNA copies^24^. However, these results were not observed in our research. Moreover, there was no significant difference in liver function between the *C*. *sinensis*/HBV co-infection group and the *C*. *sinensis* mono-infection group in this study. The reason may be that the HBV viral load of HBV-infected people in the general population is low (approximately 70% of HBV DNA < 1,000 IU/mL), most of them are in the immune tolerance stage, and liver function is at normal levels.

Among the *C*. *sinensis*/HCV co-infection, *C*. *sinensis* mono-infection and HCV mono-infection groups, the results of laboratory examinations were significantly different between the *C*. *sinensis*/HCV co-infection and *C*. *sinensis* mono-infection groups, but there was no significant difference between the C. *sinensis*/HCV co-infection and HCV mono-infection groups. This finding suggests that clonorchiasis did not aggravate the clinical manifestations of viral hepatitis. This may simply be a cross-sectional result, and it is not clear whether the long-term outcome will be the same. According to the association between eosinophil count and intensity of *C. sinensis* infection^18^, the *C. sinensis* mono-infection group had a heavier burden of *C. sinensis* than the *C. sinensis/*HCV co-infection group, but this result should be further confirmed. There was no difference in liver function indicators between the *C. sinensis/*HBV co-infection group and the *C. sinensis* mono-infection group, while the liver function indicators in the *C. sinensis/*HCV co-infection group were significantly higher than those in the *C. sinensis* mono-infection group. The reason for this difference may be that liver fibrosis was mild in the *C. sinensis*/HBV co-infection group (middle_APRI_ = 0.45, middle_FIB4_ = 1.65), while liver fibrosis in the *C. sinensis*/HCV co-infection group was severe in this study (middle_APRI_ = 0.88, middle_FIB4_ = 3.01).

The *C. sinensis* prevention and control strategies usually involve a combination of two or more measures, including health and diet safety education, environmental hygiene improvement and promotion of good personal hygiene practices, etc.^25^. Avoiding eating raw or undercooked fish and shrimp foods is the most effective way to avoid *C. sinensis* infections^26^. However, most residents in the epidemic areas find it difficult to change their habit of eating raw fish/shrimp. In our study, the population in Fuyu showed high reinfection rates after effective treatment. Therefore, people should pay more attention on the safety of freshwater fish. The distribution of freshwater fish and snails infected with metacercaria should be investigated in endemic regions, and infected water sources need to be treated in a timely manner. Finally, infected fish cannot be sold on the market^27^. The high reinfection rate suggests that exposure prevention and treatment of the second intermediate host of clonorchiasis are urgently needed. Human clonorchiasis infection rates are positively related to animal (e.g., dog, cat, etc.) infection. Animals that defecate freely lead to faster transfer of eggs into rivers and ponds, shortening the parasite’s life cycle, and finally resulting in the aggravation of the epidemic^28^. In rural areas, removing toilets and swine enclosures from fishpond areas are helpful measures for environmental improvement^27^.

In addition to focusing on the prevention of *C. sinensis*, the interaction between *C. sinensis* and its hosts and the mechanism involved in cholangiocarcinoma remain unclear. We need to further clarify the molecular mechanisms and high-risk population of *C. sinensis*-related cholangiocarcinoma, identify feasible advanced technologies^29,30^ that can screen for molecular markers for early diagnosis and disease progression monitoring, and identify drug targets.

The use of a non-random convenience sampling and self-reported risk behaviours are the key limitations of our study. The study sample may not be representative of the general population in riverside villages in Jilin Province. At the same time, the self-reporting by participants may lead to subjects' recall bias. To minimise recall bias, questions related to risk behaviour (such as "have you consumed raw fish" and "how often you consume raw fish") were included in the questionnaire. Despite these limitations, the survey demonstrated a high prevalence of *C*. *sinensis* infections in riverside villages in Jilin Province, where eating raw fish was the most important risk factor for *C*. *sinensis* transmission.

In conclusion, ten villages along the Lalin River in Desheng County are endemic areas for clonorchiasis, and the main route of infection is consuming raw freshwater fish. Co-infection with *C. sinensis* did not aggravate the clinical manifestations of viral hepatitis in this cross-sectional study, but the long-term outcomes require a follow-up survey. The high reinfection rate in our study suggests that changing dietary habits is necessary to prevent *C. sinensis* infection.

## Methods

### Study population and recruitment

In August 2017, we conducted a cross-sectional survey in 10 villages along the Lalin River in Fuyu City, Jilin Province, China^31^. The detailed survey method is similar to the HCV infection investigation we conducted in 2012^19^. First, we selected 10 villages (Beijiang, Desheng, Hejiang, Niuyingzi, Qianyang, Xiaojia, Xiaoweizi, Huandong, Linhe, and Zaixing) along the Lalin River. In the second stage, we contacted and obtained demographic information from the village chiefs. In the third stage, more than 100 village committee members and rural doctors in all 10 villages were recruited and trained for 2 days on effective publicity for the survey. They then publicised the study in their villages using recruitment cards and flyers, which was necessary for the investigators to perform cluster sampling. In the fourth stage, our research team went to each household to count the residents and distribute faecal bags. The survey was conducted for 2–3 days at each survey location. The enrolled participants were encouraged to inform their peers about the study. After screening for eligibility and obtaining informed consent, each participant completed a questionnaire. Participants who did not complete the questionnaire were excluded from the study.

### Data collection

Each participant was asked to complete a questionnaire that included information on demographic variables (age, gender and race), risk factors for *C. sinensis* infection, and history of other diseases (e.g., presence or absence of hypertension, thyroid disease, diabetes, previous history of surgery, etc.). Behavioural data regarding dietary habit use (especially eat raw fish or not and frequency of use), alcohol consumption, smoking and infection history of *C. sinensis* were also collected. People who had been confirmed to be infected with *C. sinensis* and treated with praziquantel before this cross-sectional investigation were asked to provide stool samples for testing of *C. sinensis* eggs one month after receiving treatment. Those people who were negative for 3 consecutive days one month after treatment were considered to be cured. In this investigation, people who were cured and were subsequently diagnosed with *C. sinensis* infection were considered to have a repeated infection.

### *C. sinensis* and virus detection

A modified Kato–Katz method was used to detect the *Clonorchis sinensis* eggs^32^. HBsAg, antibodies against HBsAg (anti-HBsAg), hepatitis B core antigen (anti-HBc) and hepatitis C antibody (anti-HCV) were tested in blood samples using an Abbott ARCHITECT i2000SR Immunoassay System (Abbott Laboratories; Abbott Park, IL, USA). Serum HBV DNA and HCV RNA were tested using an HBV DNA assay (Haoyuan Co., Ltd., Shanghai, China) and HCV RNA assay (Haoyuan Co., Ltd., Shanghai, China), respectively. The lower detection limits of these two kits are 50 IU/mL and 100 IU/mL, respectively. The above virological tests were completed at the clinical laboratory of the First Hospital of Jilin University.

### Statistical analysis

We conducted statistical analysis using SPSS software package 18.0 (SPSS Inc., Chicago, IL, USA). Continuous variables were expressed as medians and interquartile ranges or means and standard deviations, as appropriate, and were compared using Student’s t-test or the Mann–Whitney U-test. Categorical variables were compared using the chi-square test or Fisher’s exact test. Variables with statistical significance (*P* ≤ 0.05) in the univariate analysis were considered for inclusion in the multivariable logistic regression model. Then, we performed backward stepwise logistic regression to screen independent risk factors retained in the final model. A *P*-value ≤ 0.05 was considered statistically significant; odds ratios (ORs) with 95% confidence intervals (95% CIs) are presented to demonstrate the strength and direction of these associations.

### Ethical considerations

The study was approved by the Ethics Committee at the First Hospital of Jilin University (2017-023), Changchun, China. Each participant signed an informed consent form before participating in the survey. During the investigation, we protected participants from potential ethical violations and complied with data confidentiality agreements. Data collection was conducted at the township health centre. During the questionnaire process, the interviewee and interviewer were required to be present at the same time, and only the two of them were present to ensure the maintenance of confidentiality. Those agreeing to take part in the study were asked to have their blood collected for further evaluation of anti-HCV and anti-HBV activities, always in the presence of the researcher. The collection of tissues and inclusion of patients were approved by the Ethics Committee at the First Hospital of Jilin University (2017-023), Changchun, China. Moreover, this study was in accordance with the regulations set by Chinese law for the use of human material for research^33^.

## Data Availability

The datasets generated and/or analysed during the current study are available from the corresponding author upon request.
